# Clinical Features and Surgical Outcomes of Osteochondroma of the Spine

**DOI:** 10.5704/MOJ.2303.014

**Published:** 2023-03

**Authors:** Y Sakai, H Nakashima, T Takatsu, S Imagama

**Affiliations:** 1 Department of Orthopaedic Surgery, Ichinomiya Orthopaedic Clinic, Ichinomiya, Japan; 2 Department of Orthopaedic Surgery, Gifu Prefectural Tajimi Hospital, Tajimi, Japan; 3 Department of Orthopaedic Surgery, Medical Cooperation Rokujukai Goto Orthopaedic Clinic, Tsushima, Japan; 4 Department of Orthopaedic Surgery, Nagoya University Graduate School of Medicine, Nagoya, Japan

**Keywords:** osteochondroma, spine, exostosis, surgical excision

## Abstract

**Introduction:**

Spinal osteochondroma is rare. The purpose of this study is to examine the clinical characteristics and surgical treatment outcomes of 11 patients with spinal osteochondroma.

**Materials and methods:**

The study included 11 patients with spinal osteochondroma. In these patients, we examined the onset level, onset site, initial symptoms, surgical procedure, outcomes and complications.

**Results:**

Of the 11 patients, 9 presented with solitary tumours, and 2 had multiple. The mean post-operative observation period was six years and two months. The onset level was the cervical spine in eight patients, thoracic in two, and lumbar in one. The most common onset site was the posterior elements. The initial presentation was myelopathy in seven patients, radiculopathy in two, neck pain in one and feeling of mass in one. All patients underwent excision of the tumour, and depending on the tumour onset site, additional posterior or anterior decompression with or without fusion was performed. There was no recurrence in all patients. Intra-operative complications included dura tear and oesophageal injury in one patient with cervical onset, while post-operative complications included C5 palsy in one patient.

**Conclusions:**

In this study, surgical excision for osteochondroma of the spine were excellent with no recurrence of the tumour.

## Introduction

Osteochondroma (exostosis) is known to be the most common benign bone tumour, accounting for 10–15% of all bone tumours and 20–50% of all benign bone tumours^1^. These bone tumours generally arise in the metaphysis of long bones, presenting mature bone tissue that is continuous with the bone marrow and a cartilage cap. Spinal onset is rare, and less than 5% of solitary osteochondromas develop in the spine^2-5^.

In the present study, we examined the clinical characteristics and treatment outcomes of 11 patients with spinal osteochondroma who underwent surgical treatment and report our findings.

## Materials and Methods

The study included 11 patients with spinal osteochondroma (5 men and 6 women) who underwent surgical treatment from 1997 to January 2017. Of the 11 patients, 9 presented with solitary tumours, and 2 had multiple tumours. The mean age at the time of surgery was 43.4 (13–63) years, and the mean post-operative observation period was 3 years and 9 months, and the median was 6 years and 2 months (range: 1 year and 3 months to 12 years and 3 months). In these patients, we examined the onset level, onset site, initial symptoms, surgical procedure, Japanese Orthopaedic Association (JOA) score for cervical myelopathy^6^, and low back pain^7^ (evaluated according to a maximum score of 11 points for the thoracic spine, excluding upper extremity function of the cervical myelopathy treatment determination criteria) (JOA score), improvement according to the Hirabayashi method^8^, outcomes, and complications.

This study was approved by our institutional review board and written informed consent was obtained from all patients for the publication of this paper and any accompanying images.

## Results

The onset level was the cervical spine in 8 patients, thoracic spine in two patients, and lumbar spine in one patient. The onset site was the lamina in five patients, vertebral body in one patient, facet joint in one patient, vertebral body and lamina in one patient, lamina and pedicle in two patients, and pedicle and head of the rib in one patient. The initial presentation was myelopathy in seven patients (cervical spine in five patients and thoracic spine in two patients), radiculopathy in two patients (cervical spine in one patient and lumbar spine in one patient), neck pain (cervical spine in one patient), and feeling of mass (cervical spine in one patient).

For the surgical procedure, all patients underwent excision of the tumour, and depending on the tumour onset site, additional posterior decompression was performed in five patients, posterior decompression with fusion in three patients, and anterior fusion with posterior decompression and fusion in three patients.

JOA scores changed from a mean of 14.4 points pre-operatively to 15.9 points post-operatively for the cervical spine, with a mean improvement rate of 57.7%; 6.5 points pre-operatively to 9.5 points post-operatively for the thoracic spine, with a mean improvement rate of 66.7%; and 23 points pre-operatively to 27 points post-operatively for the lumbar spine, with an improvement rate of 66.7% (Table I).

**Table I:** 

Case	Age (yrs) / Sex	Lesion type	Tumour level	Tumour site	Localisation	Initial presentation	Surgical procedure	Pre-operative JOA score (points)	Post-operative JOA score (points)	Post-operative follow-up periods (months)	Complication
1	62/M	solitary	C 1	lamina	intra-canal	myelopathy	tumour excision, posterior decompression	15	17	18	none
2	13/M	multiple	C 2	lamina	intra-canal	feeling of mass	tumour excision, posterior decompression	17	17	62.4	none
3	62/M	solitary	C 4	vertebral body	intra-canal	neck pain	tumour excision, posterior decompression with fusion	17	13	96	C5 palsy
4	63/F	solitary	C 5	lamina	intra-canal	myelopathy	tumour excision, posterior decompression with fusion	13	15	15	none
5	49/F	solitary	C 5	vertebral body / lamina	intra-canal	myelopathy	tumour excision, anterior fusion and posterior decompression with fusion	13	16	82.4	dura tear and oesophageal injury
6	43/F	solitary	C 6	lamina	intra-canal	radiculopathy	tumour excision, posterior decompression	14	17	27.2	none
7	45/M	solitary	C 7	lamina	intra-canal	myelopathy	tumour excision, posterior decompression	13	16	45.3	none
8	17/F	solitary	C 7	facet joint	intra-canal/extra-canal	myelopathy	tumour excision, anterior fusion and posterior decompression with fusion	13	16	148	none
9	44/F	solitary	Th 9	Pedicle / lamina	intra-canal	myelopathy	tumour excision, posterior decompression with fusion	3	8	32.3	none
10	35/M	multiple	Th 10	pedicle/head of the rib	intra-canal/extra-canal	myelopathy	tumour excision, posterior decompression with fusion	10	11	92.4	none
11	51/F	solitary	L 4	pedicle/lamina	intra-canal/extra-canal	radiculopathy	tumour excision, anterior fusion and posterior decompression with fusion	23	27	24	none

Abbreviations - C: cervical spine, Th: thoracic spine, L: lumbar spine, JOA score: Japanese Orthopaedic Association score

In all patients, there was no recurrence observed during the follow-up observation period. Intra-operative complications in patients with cervical spine onset included dura tear and oesophageal injury in 1 patient, while post-operative complications included C5 palsy in one patient.

**Fig. 1:**
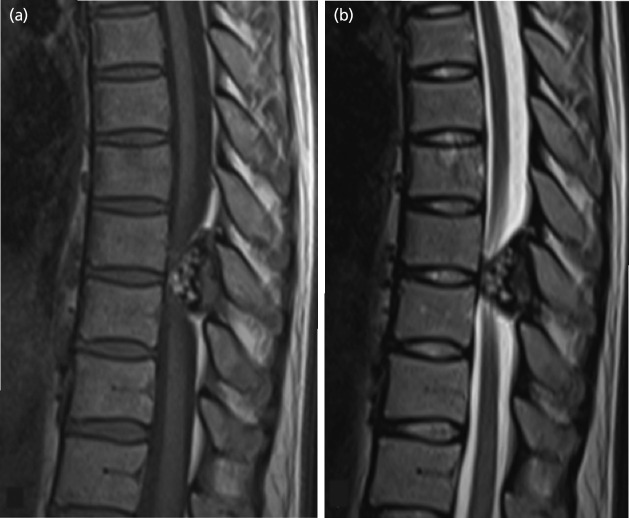
Case 9. MR images demonstrating a mass protruding into the spinal canal on the abdominal side of the lamina of Th9/10 level. (a) sagittal T1-weighted image, (b) sagittal T2-weighted image.

**Fig. 2:**
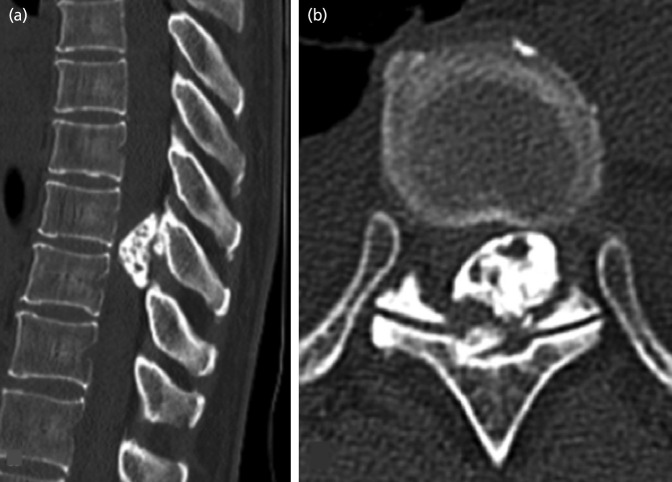
Case 9. Plain CT showing osseous lesion in continuity with the lamina and protrusion into the spinal canal. (a) sagittal image, (b) axial image.

**Fig. 3:**
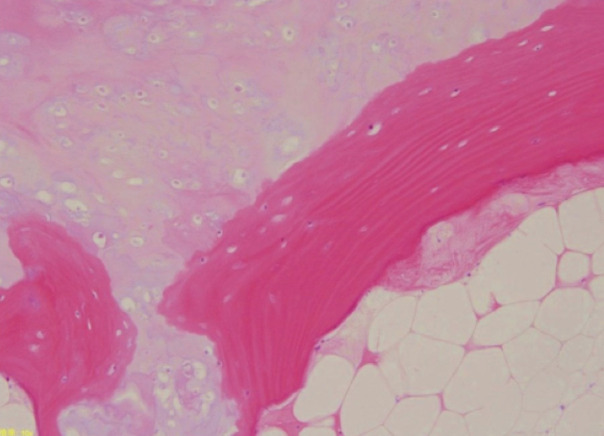
Case 9. Pathological examination showing bone tissue with low grade dysplasia under cartilage tissue (Hematoxylin and eosin stain; 100 x magnification).

For the case presentation, Case 9: the case subject was a 44-year-old woman with no medical history. She noticed numbness in the lower extremities at 32 weeks of pregnancy and developed buckling of the knees when walking at 33 weeks of pregnancy. Therefore, at 34 weeks of pregnancy, she consulted our department. Reduced thermal nociception below the level of the 9th thoracic vertebra and spastic partial paralysis of the bilateral lower extremities were observed. Magnetic resonance imaging (MRI) revealed a 11 × 9 × 26mm mass protruding into the spinal canal on the abdominal side of the lamina of the 9th/10th vertebra. The mass margin presented low signal intensity on both T1- and T2-weighted imaging, while part of the inner portion of the mass showed high signal intensity. The spinal cord was compressed, and intramedullary signal intensity change was observed on T2-weighted imaging (Fig. 1). Plain computed tomography (CT) revealed an osseous protrusion in continuity with the lamina and progressing into the spinal canal (Fig. 2). Based on the imaging findings, it seemed to be osteochondroma arising from the lamina; thus, at 35 weeks of pregnancy, Caesarean section was performed, and the following week, tumour resection and laminectomy was performed under general anaesthesia. The pathological findings of the resected specimen revealed bone tissue with low-grade dysplasia under cartilaginous tissue; thus, osteochondroma was diagnosed (Fig. 3). The paralysis gradually improved, and presently, three years after surgery, the subject is capable of jogging, and there is no recurrence noted on imaging.

**Fig. 4:**
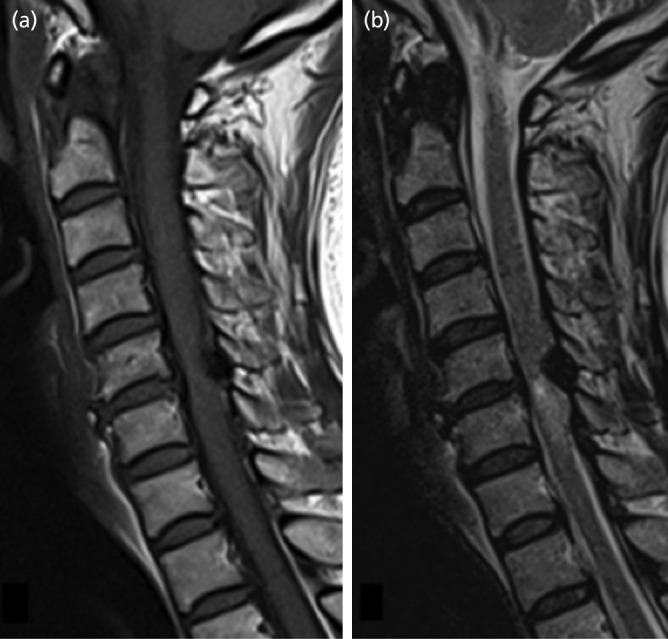
Case 4. MR imaging demonstrating osseous lesion protruding into the spinal on the abdominal side of the lamina of the C5. (a) sagittal T1-weighted image, (b) sagittal T2-weighted image.

**Fig. 5:**
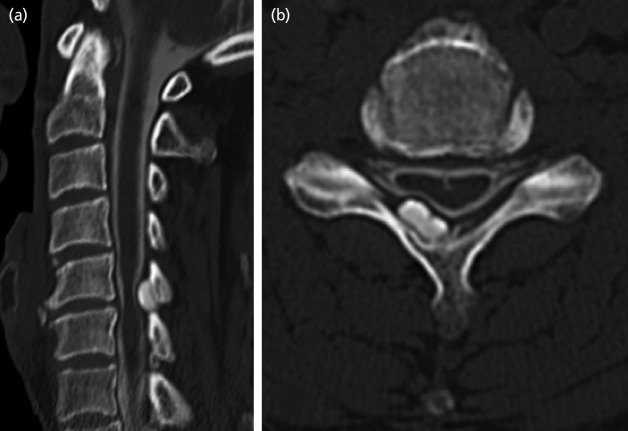
Case 4. CT after myelography showing an osseous lesion in continuity with the lamina and protruding into the spinal canal, with compression of the dural sac. (a) sagittal image, (b) axial images.

Case 4: The case subject was a 63-year-old woman. She consulted our department after noticing numbness on the left hand and weakness of the right lower extremity. She complained of numbness in the region of the C6 nerve, and impaired manual dexterity with mild gait impairment was observed. The tendon reflex was accentuated distal to the triceps reflex, and a pathological reflex was also observed. On MRI, a 9 × 4 × 8mm tumour protruding into the spinal canal was observed on the abdominal side of the lamina of the C5 vertebra. The tumour showed homogeneous low signal intensity on both T1- and T2-weighted imaging. The spinal cord was compressed, and on T2-weighted imaging, intramedullary signal intensity change was observed (Fig. 4). CT after myelography revealed an osseous protrusion in continuity with the lamina and protruding into the spinal canal, with compression of the dural sac (Fig. 5). Based on the imaging findings, it seemed to be osteochondroma arising in the lamina; thus, tumour resection and posterior decompression with fusion was performed under general anaesthesia. Osteochondroma was diagnosed based on the pathological findings of the resection specimen. Presently, two years after surgery, there is residual numbness; however, the subject is able to perform activities of daily living, and there has been no recurrence observed on imaging.

## Discussion

Osteochondroma (exostosis) is the most common benign tumour, presenting as solitary tumours (90% of cases) and multiple tumours (10% of cases) in the form of hereditary multiple exostosis (HME)^9,10^. Osteochondroma arising from the spine is relatively rare, accounting for 1–4% of solitary osteochondromas and 1–9% of HME^2-5,9,10^. It has been reported that the most common onset site is the cervical spine (~50% of cases), particularly at the C2 vertebra, followed by the thoracic and lumbar spine^1,2,5,9^. Furthermore, it is said that solitary osteochondroma commonly affects the cervical spine, whereas HME commonly affects the thoracic and lumbar spine^4,9,10^. Our patients also presented similar results. In the thoracic spine, the Th8 vertebra is most commonly affected, followed by the Th4 vertebra^1,2^. Among our patients with onset of the thoracic spine, onset was at the lower levels of the thoracic spine affecting the Th9 and Th10 vertebra. The reason that onset commonly affects the cervical spine is said to be attributed to great mobility, which places minute external force and stress on the epiphyseal cartilage, which leads to its dislocation^2,9,11^.

With regard to onset site, tumours most commonly develop in the posterior elements and protrude dorsally, whereas anterior onset, such as of the vertebral body, is rare^1,2,11^. The fact that there is rare onset of osteochondroma in vertebral bodies can probably be explained by the absence of an epiphyseal plate^2^. In contrast, the reason that onset is common in the posterior elements of the spine is attributed to the fact that there are many secondary ossification centres (e.g., spinal process, transverse process, and condylar processes) and that osteochondromas develop from the excessive cartilaginous tissue of such secondary ossification centres^2,11^. Furthermore, tumours protruding dorsally from posterior elements (lamina and spinal process) are typically large and found as tumours that can be palpated, have superficial deformation in young individuals, and lack neurological symptoms^1^. In our patients, onset in the posterior elements, such as the lamina and facet joint, accounted to be about 80%. Osteochondroma progressing into the spinal canal, even if small, can cause concurrent neurological symptoms. Spinal osteochondroma causing myelopathy is considered rare^2,9^, with reports indicating an incidence of 0.5–1% in solitary lesions and approximately twofold in multiple lesions^2^, whereas a somewhat high incidence of 27.2–34% has also been reported in solitary spinal osteochondroma^1,3^. Of our 11 patients, 7 (63.6%) presented with myelopathy. This is thought to be attributed to persistent spinal cord compression when the tumour continues to grow even after completion of bone growth in spinal osteochondroma^2,3^. Furthermore, in our patients, the age of patients who developed myelopathy was older, at 35–63 years, with onset also found in elderly individuals, and it is possible that age-related degenerative changes in the spine contributed to myelopathy onset^3,12^.

In the diagnosis of spinal osteochondroma, CT is particularly useful. Typical CT findings include the following: (1) round tumour with distinct border, (2) bone-like concentration with diffuse calcification, (3) paravertebral, dumbbell type, or eccentric intraspinal onset, and (4) continuity of the lesion with the cortical bone and medullary space^3,5^, which were similar to the findings in our patients. MRI findings include low signal intensity on T1-weighted imaging, reflecting a cartilage cap on the osteochondroma with cartilage rich in water content, and high signal intensity on T2-weighted imaging, while the marrow part presents high signal intensity on both T1- and T2-weighted imaging due to rich fatty content^1,2,13^. Furthermore, it is important to evaluate the thickness of the cartilage cap, and lesions with a cartilage cap exceeding 1 or 3cm have an increased risk of malignant transformation^2,4,5,14^.

Surgery is indicated for tumours developing within the spinal canal; those compressing the spinal cord, cauda equina, and nerve root with neurological symptoms; those suspected of malignant transformation; and those with pain and restricting range of motion^3^. For osteochondroma, it is important that the cartilage cap is completely surgically removed to prevent recurrence^2,3,14^. Tumours generally develop in the posterior elements; therefore, full or partial laminectomy, depending on the size of the lesion and removal of the spinous process by posterior approach is often used for marginal resection; however, depending on the onset site of the tumour, an anterior or anteroposterior approach will be needed^2,3^. Furthermore, when the facet joint is resected, spinal fusion will be required^14^.

The rate of recurrence following osteochondroma excision is 1.3–8%^2,3,11^, and the mean duration until recurrence ranges from 1 to 26 years^2^. Furthermore, the incidence of malignant transformation is 1–3% in solitary osteochondroma^2,3^ and 10–12% in HME^2,11^. In our patients, during the mean post-operative follow-up observation period of six years and two months, there was no recurrence observed; however, we believe that, in the future, long-term follow-up observation is needed.

## Conclusions

Osteochondroma is relatively common bone tumour, accounting for 20-50% of all benign bone tumours, but occurs infrequently in the spine accounting for less than 5% of solitary osteochondromas. After examining the clinical characteristics and treatment outcomes of 11 patients with spinal osteochondroma who underwent surgical treatment, we found that these lesions caused neurologic symptoms including myelopathy (63.6%) and radiculopathy (18.2%). All patients underwent tumour excision including cartilaginous cap and no recurrence was observed. When tumour excision is performed adequately, the outcomes are excellent with very low recurrence rates.
